# The Effect of Anti-aging Peptides on Mechanical and Biological Properties of HaCaT Keratinocytes

**DOI:** 10.1007/s10989-017-9648-7

**Published:** 2017-11-16

**Authors:** Tomasz Kobiela, Małgorzata Milner-Krawczyk, Monika Pasikowska-Piwko, Konstancja Bobecka-Wesołowska, Irena Eris, Wojciech Święszkowski, Ida Dulinska-Molak

**Affiliations:** 1 Institute of Biotechnology, Faculty of Chemistry, Warsaw University of Technology, Noakowskiego 3, 00-664 Warsaw, Poland; 2 Dr Irena Eris Cosmetic Laboratories, Centre for Science and Research, Armii Krajowej 12, 05-500 Piaseczno, Poland; 3 Faculty of Mathematics and Information Science, Warsaw University of Technology, ul. Koszykowa 75, 00-662 Warsaw, Poland; 4 Faculty of Materials Science and Engineering, Warsaw University of Technology, Wołoska 141, 02-507 Warsaw, Poland

**Keywords:** Atomic force microscopy, HaCaT, Cell stiffness, Cytoskeleton, Anti-aging peptides

## Abstract

Atomic force microscopy (AFM) and fluorescence microscopy was applied to determine the influence of the anti-aging peptides on the morphology and the mechanical properties of keratinocytes. Immortalized human keratinocytes (HaCaT) were treated with two anti-aging bioactive peptides: Acetyl Tetrapeptide-2 and Acetyl Hexapeptide-50 (Lipotec). The AFM measurement of the keratinocyte stiffness were carried after 48 h exposure at an indentation depth of 200 nm. AFM analysis showed increase of the cell stiffness for cells treated with Acetyl Tetrapeptide-2 (P1) in concentration range. Acetyl Hexapeptide-50 (P2) at concentration of 0.05 µg/ml also increased the stiffness of HaCaT cells but at higher concentrations 0.5 and 5 µg/ml cell stiffness was lower as compared to untreated control. Fluorescence microscopy revealed remodeling of actin filaments dependent on the concentration of P2 peptide. The mechanical response of HaCaT cells treated with P2 peptide corresponds to change of transcription level of ACTN1 and SOD2 which activity was expected to be modulated by P2 treatment.

## Introduction

In response to still growing demands, the researchers working in pharmaceutical and cosmeceutical fields are developing new, more efficient, bioactive and biocompatible anti-aging compounds. It is known that small molecules, with molecular weight less than 500 Da, are able to penetrate skin permeability barrier of the *stratum corneum* (Vecchia and Bunge 2003). The most successfully and widely used in cosmetic products are topical peptides categorized by Gorouhi and Maibach as signal peptides, enzyme inhibitory peptides, neurotransmitter-inhibitory peptides and carrier peptides (Gorouhi and Maibach 2009). First group - signal peptides generally stimulate protein production including the components of extracellular matrix (ECM) like collagen, elastin, glycosaminoglycans and fibronectin. It has been demonstrated that fibroblasts connected with ECM network provide support for the epidermis and are responsible for elastic properties of the skin connective tissue (Metcalfe and Ferguson 2007; Sorrell and Caplan 2004). Signal peptides stimulating activity of fibroblasts resulting in ECM collagen production result in firmer and younger look of the skin. Mechanical properties of the skin change not only with aging but also under influence of various external factors like UV irradiation and environmental chemical pollution (Sobiepanek et al. 2016). This leads to a disturbance in synthesis of collagen and other structural ECM and cellular proteins causing the loss of skin firmness and elasticity.

The evaluation of mechanical properties of living cells has become possible with the development of local measurement techniques such as atomic force microscopy (AFM). Since its discovery, distinct cell types have been investigated e.g. blood or cancer cells in various human pathologies (Dulinska et al. 2006; Lim 2006; Pogoda et al. 2012; Suresh 2007) or even carbon nanotubes effect on the mechanical properties of bovine articular chondrocytes and human bone marrow-derived mesenchymal stem cells was studied (Dulinska-Molak et al. 2013, 2014a). Also skin fibroblasts and keratinocytes have been analyzed (Berdyyeva et al. 2005; Dulinska-Molak et al. 2014b; Lulevich et al. 2010; Schulze et al. 2010). Berdyyeva and coworkers showed that the human foreskin epithelial cells became significantly more rigid during aging (Berdyyeva et al. 2005). These results are in agreement with report of Dulińska-Molak where fibroblasts isolated from 60-year old donor were much more rigid compared to cells isolated from younger subjects (Dulinska-Molak et al. 2014b). Recently, the decrease of the cell stiffness of living epidermal keratinocytes treated with sodium lauryl sulphate was presented (Kobiela et al. 2013).

Keratinocytes are the major cell type present in the epidermis, which are capable of producing cellular and ECM structural proteins i.e. fillagrin, involucrin, loricrin and keratins. It has been shown that remodeling of the keratin cytoskeleton is crucial for cell–cell and cell-matrix adhesion, a key process for cells motility during wound healing or inflammation (Seltmann et al. 2013). Although epidermal cells are important for the formation of the physical barrier against environmental factor, as well as for skin condition, their biomechanical properties are so far only partially elucidated. For that reason the effect of bioactive peptides on skin cells elastic properties is essential to be understood. We investigated the influence of two anti-wrinkle bioactive peptides on mechanical properties of HaCaT keratinocytes. Peptide 1 (P1, Acetyl Tetrapeptide-2) is known as a stimulator of structural skin elements including collagen and elastin. Peptide 2 (P2, Acetyl Hexapeptide-50) simulates action of transcription factor FOXO3a, responsible for protection of DNA and maintenance of cellular homeostasis. We showed that HaCaT keratinocytes exhibit a change in elasticity after anti-wrinkle peptides treatment in vitro. Moreover, our results indicate change of genes expression crucial for activity of both peptides.

## Materials and Methods

### Cell Lines

HaCaT keratinocyte line (Deutsches Krebsforschungszentrum Stabsstelle Technologietransfer Heidelberg, Germany) was cultured in DMEM (Sigma, USA) medium supplemented with 10% fetal calf serum (FCS, Life Technologies, USA), 10 mM HEPES (Life Technologies, USA), 2 mM L-glutamine (Life Technologies, USA), and antibiotics (100 U/ml penicillin, 0.25 µg/ml streptomycin sulfate, Life Technologies, USA). Cells were grown at 37 °C in humidified atmosphere with 5% CO_2_. Cells at 80–90% confluency were collected using trypsin–EDTA solution (0.05%; Life Technologies, USA), centrifuged (1500 rpm, 10 min), diluted ten times in growth medium and seeded on glass coverslips (18 mm ×T18 mm, Menzel Gläser, Germany), placed in 6-well culture dishes and incubated at 37 °C, 5% CO_2_ for 48 h.

### Peptide Treatment

Cultured cells were treated with solution (water: caprylyl glycol or water: butylene glycol) of anti-wrinkle peptides (0.05% of active ingredient, Lipotec, Spain): peptide 1 (P1, Acetyl Tetrapeptide-2, Lipotec) and peptide 2 (P2, Acetyl Hexapeptide-50, Lipotec) were dissolved in complete DMEM medium in a range of concentrations from 0.05 to 50 µg/ml. After removal of the culture medium, HaCaT cells were exposed to the peptides solutions. Controls containing culture medium were included. Plates were incubated at 37 °C, 5% CO_2_ for 48 h for cell viability, gene expression analysis, AFM measurements and visualization of actin filaments.

### Cell Viability Assay

The MTT [3-(4, 5-dimethylthiazol-2-yl)-2, 5-diphenyltetrazolinum bromide] assay was used to assess cell viability according to the following protocol. MTT (Sigma-Aldrich, USA) was dissolved in DMEM (Cytogen, Germany) at the concentration of 0.5 mg/ml. After cell treatment with the investigated peptides, medium was replaced by 100 µl/well of the MTT solution followed by incubation for 2 h at 37 °C, 5%CO_2_. Solubilization of formazan product was performed by addition of 100 µl/well of isopropanol and shaking for 15 min at room temperature (RT). The sample absorbance was determined at 595 nm wavelengths in a microplate reader (Synergy H4, Biotek) The results are expressed as a percent of untreated control and are an average of three experiments.

### RNA Purification and Q-PCR Amplification

The HaCaT cells were cultured for 48 h in the presence of peptides. Total cellular RNA was extracted from cells with PureLink RNA Mini Kit (Life Technologies). Subsequently, five micrograms of isolated RNA was used for cDNA synthesis using First Strand cDNA Synthesis kit (Novagen) according to the manufacturer’s protocol. In the cells treated with the P1 expression of *ACTN1, ITGB4, COL17A1* was checked. In the cells treated with the P2 expression of *ACTN1, FOXO1* and *SOD2* was checked. Genes expression quantification was performed by real-time PCR (Q-PCR) on a 7500 Real Time PCR System (Applied Biosystems). All PCR primers (Table 1) were designed using software Primer Quest (http://eu.idtdna.com/PrimerQuest/Home/Index). Reactions were carried out in a reaction mixture containing 1× concentrated mixture SensiFast Low ROX (Bioline) with SYBR Green, primers forward and reverse (100 µM each), the cDNA template, and nucleases free water (Thermo Scientific) to a final volume of 10 µl. Reaction protocol was as follows: initial denaturation at 95 °C for 3 min., followed by 45 cycles of denaturation (95 °C for 15 s.), hybridization and elongation (60 °C for 1 min). The relative expression of each gene were calculated by the modified method of ΔCt in relation to the geometric mean of two reference genes Ct (Vandesompele et al. 2002). The experiment was conducted in two biological and three analytical replications.

**Table 1:** Primers used in Q-PCR amplification

Gene	Name	Sequence (5′→3′)	Tm (°C)	Amplikon length (bp)
*PMM1* (ref.)	hPMMaF	CGCCTTCCTGCAGAAGCTAC	59	176
	hPMMaR	TCTGCTTGGAGAGCAGTCGTC	59	
*GUSB* (ref.)	hGUSBaF	CAGGGTTTCACCAGGATCCAC	60	176
	hGUSBaR	TTTATTCCCCAGCACTCTCGTC	59	
*ACTN1*	hACTN1aF	AGAAATCGTGGATGGGAATG	60	190
	hACTN1aR	GCCATCCTTCCAGCTTATG	60	
*COL17A1*	hC17A1aF	GTACATGCAGAGTGACAGTATTA	60	190
	hACTN1aR	AGAGATGGAGGACGAGAAC	60	
*FOXO1*	hFOXO1aF	TGACTTGGATGGCATGTTC	60	190
	hFOXO1aR	GTGTAACCTGCTCACTAACC	60	
*ITGB4*	hITGB4aF	CTGGAAGGTCACCAACAAC	60	192
	hITGB4aR	TAGACCTCGTTCAGGTTCTC	60	
*SOD2*	hSOD2aF	TCCAGGCAGAAGCACAG	61	140
	hSOD2aR	TTCTCCTCGGTGACGTTC	61	

### Atomic Force Microscopy

Measurements of the HaCaT cells topography and stiffness were performed using a commercial microscope (XE120 model, Park Scientific Instruments, South Korea). The gold-coated silicon nitride cantilevers (MLCT, Bruker, USA) with a spring constant of 0.01 N/m were applied both for topography imaging and elasticity measurements. For all images we started from the same values of scan parameters (scan rate was 1 Hz and set point was set to 0.5 nN) however in each case final optimization were performed. Force curves were collected from randomly chosen cells, localized by a top-view optical camera integrated with the AFM and imaged before elasticity measurements. Regions around cell center were randomly selected for the measurements and at each region, a grid of 4 × 4 points was created. Force curves were recorded at the scan velocity equaled to 9 µm/s.

### Actin Filaments Visualization

The arrangement of actin filaments was visualized using Alexa Fluor 488 Phalloidin staining. The HaCaT keratinocytes grown on coverslips and treated with the different concentration of each peptide (see “Peptide Treatment” section) were fixed with 3.7% paraformaldehyde/PBS for 10 min at RT, rinsed with PBS (10 min, RT), permeabilized with 0.1% Triton X-100/PBS (5 min, RT) and rinsed with PBS (10 min, RT). Subsequently, the actin filaments were stained using the phalloidin labeled with Alexa Fluor 488 (1:200, Life Technologies, USA) for 30 min at RT and washed by 10 min incubation in PBS at RT. The wet coverslips were placed on the microscope slide and closed with nail varnish and left to dry for 2 h. The cells were imaged using Olympus IX71 (Olympus, USA) fluorescent microscope equipped with the halogen lamp.

### Elastic Modulus of Keratinocytes

Determination of the elastic modulus was based on a subtraction of two force curves: the calibration curve recorded on a hard surface (in our case, it was a part of glass coverslip without cells) and the other on a given cell, as described previously (Kobiela et al. 2013).

The obtained *force versus indentation curve* can be fit with the Sneddon extension of the Hertz model (Sneddon 1965) assuming that the AFM tip is an infinitely stiff indenter of conical geometry. Then, the elastic modulus can be calculated according to the equation:

1 $$F(\delta )=\frac{{2 \cdot E^{\prime} \cdot \tan \alpha }}{\pi } \cdot \Delta {\delta ^2}$$

where *α—*is the open angle of the cone (of the AFM tip), *E’*′—is the reduced elastic modulus, which considers both the cell and AFM cantilever stiffness linked in series. Since the elastic modulus of the AFM cantilever is much larger than that for cells, the *E*′ can be re-written as follows:

2 $$E'=\frac{{{E_{cell}}}}{{1 - \mu _{{cell}}^{2}}}$$

where *µ*_*cell*_ is the Poisson coefficient set to 0.5 since cells can be treated as an incompressible material (Lekka et al. 1999).

### Statistical Analysis

The statistical analysis was performed using R software (http://www.R-project.org). The significance of differences between two compared parameters was assessed by Student t-test, Welch test and Wilcoxon test, depending on the probability distribution of the analyzed data. To compare three groups one way ANOVA or Kruskal- Wallis test was used. We considered p values < 0.05 significant.

## Results and Discussion

### Cell Viability

**Fig. 1:**
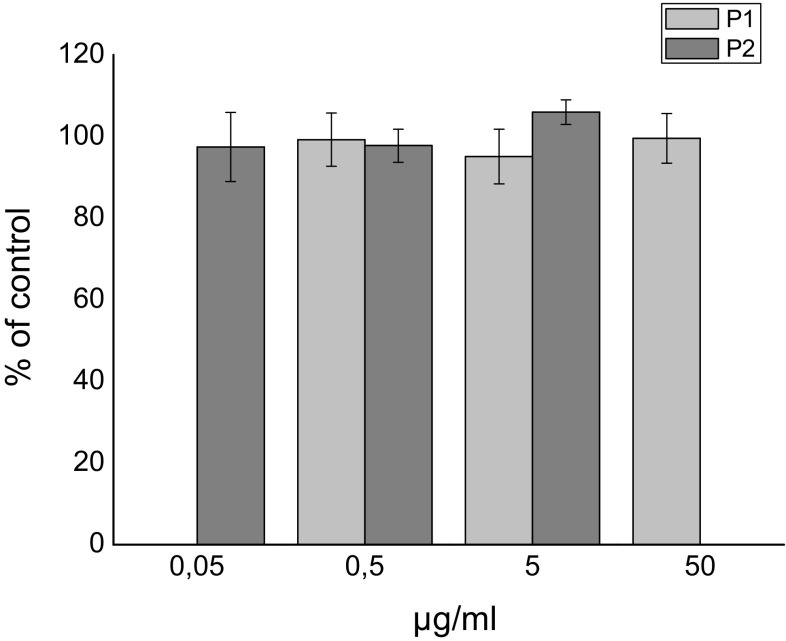
The viability of HaCaT cells after 48 h treatment with P1 acetyl-tetrapeptide-20 (light gray) and P2 acetyl-hexapeptide-50 (dark gray). Data were normalized to mean control value. The results are expressed as mean values ± standard deviations

The cytotoxicity of the investigated peptides for HaCaT cells analyzed by MTT assay after 48 h treatment with various peptide concentrations is shown on Fig. 1.

The experiment showed that both peptides are non-toxic for the cells in investigated concentration range and treatment time (Fig. 1).

### Transcriptional Profiling of Keratinocytes in Response to P1 and P2 Peptides

**Fig. 2:**
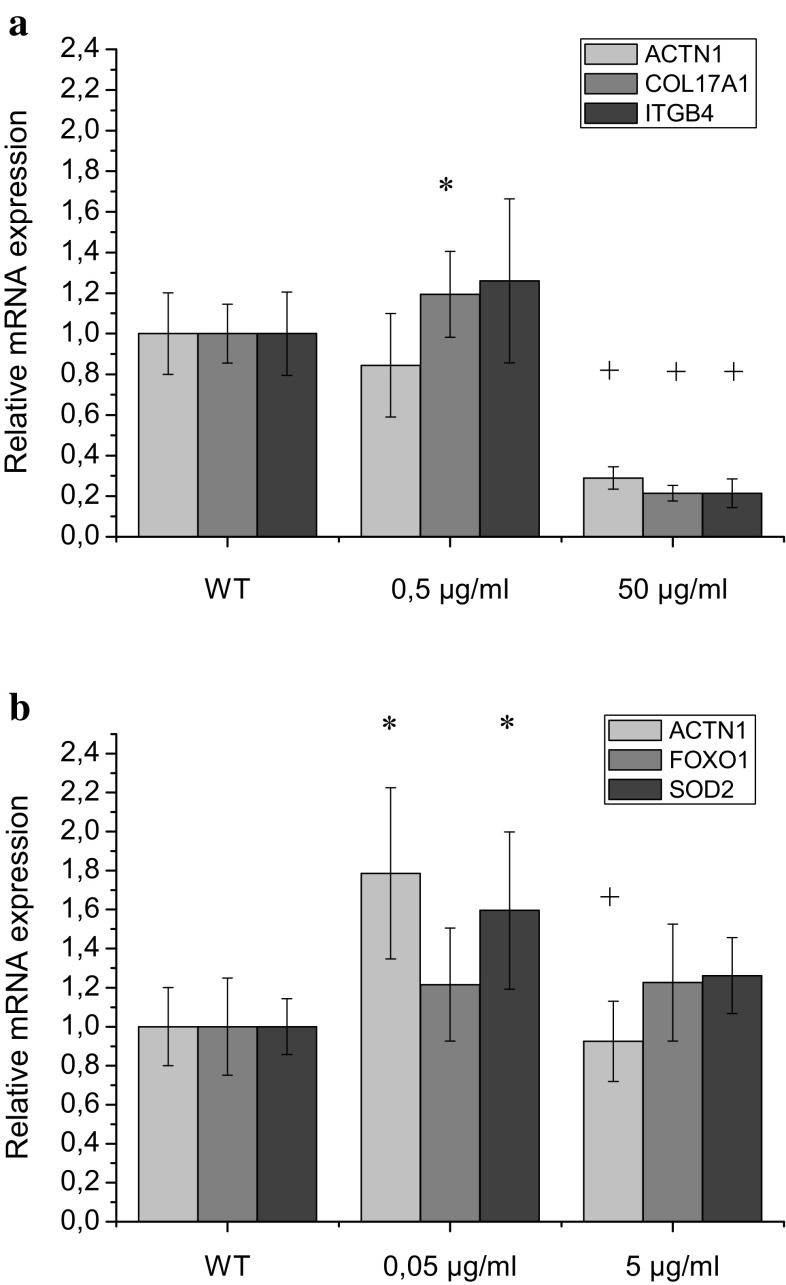
Relative mRNA expression of ACTN1, ITGB4, COL17A1, FOXO1 and SOD2 in response to P1 acetyl-tetrapeptide-20 (**a**) and P2 acetyl-hexapeptide-50 (**b**) treatment of HaCaT cells after 48 h of incubation with the peptides. ACTN1 (light gray), COL17A1/FOXO1 (gray), ITGB4/ SOD2 (dark gray). Data were normalized to mean control value (WT- cells without treatment). The results are expressed as mean values ± standard deviations. By ‘*’ we denote the cases where the gene expression was significantly different from control (p < 0.05) and by ‘+’ we denote the significant difference between the gene expression in cells treated with peptide in the lowest and the highest concentration (p < 0.05)

The multi-faceted process of skin aging and wrinkles generation is still under investigations, but the integral part of it is modulation of cellular mechanosensitivity and response to external tension like gravity (Piérard et al. 2010). The proposed mechanism of the cellular response to changes in mechanical conditions and cell mechanosensitivity homeostasis is defined by the state of the actin cytoskeleton which is directly related to the changes in alpha-actinin-1 and alpha-actinin-4 level in the cytoplasmic and membrane fractions (Ogneva 2013). Therefore, to monitor the cell mechanosensitivity response to the P1 and P2 peptides we have taken monitoring of mRNA level of alpha-actinin-1 (ACTN1). Another genes selected for expression monitoring were connected with suggested by the peptide producer—Lipotec mechanism of investigated peptide activity. Therefore, mRNA level of collagen type XVII alpha 1 (COL17A1) and integrin beta 4 (ITGB4) following P1 treatment was monitored. After P2 treatment the level of mRNA of forkhead transcription factor 1 (FOXO1) and manganese-dependent superoxide dismutase 2 (SOD2) was verified. Both peptides were incubated 48 h with keratinocyte before mRNA isolation and Q-PCR amplification. The effect of P1 and P2 on the expression of investigated genes is demonstrated on Fig. 2.

In the case of the lowest P1 concentration (0.5 µg/ml) statistically significant (p < 0.05) up-regulation of *COL17A1* was observed. Along with the increase of P2 concentration strong down-regulation (p < 0.05) of the expression of three tested genes (*ACTN1, COL17A1* and *ITGB4*; Fig. 2a) was noticed. Such down-regulation of *ACTN1* and *ITGB4* suggest dysfunctional actin filament bundles and as a consequence lower mechanical properties of treated with P1 kertainocytes. Taking into account that ACTN1 and ITGB4 participate in Focal Adhesion formation and interface between the cell and Extracellular Matrix, and that COL17 is a part of multiprotein hemidesmosome complex that mediates adhesion of epithelial cells to the underlying basement membrane, it seems that higher doses of those peptide do not influence positively on resistance of the cells neither to gravity nor aging.

On the contrary P2 keratinocyte treatment appear to be much less invasive to cell homeostasis. The significant up-regulation of *ACTN1* and *SOD2* (p < 0.05) in cells treated with P2 0.05 µg/ml was observed. The expression level of *FOXO1* gene was unchanged (p > 0.05). The up regulation of *ACTN1* suggests positive contribution of P2 to the cell mechanical properties. In turn, up-regulation of *SOD2* confirms positive role of P2 in protection of the cell against oxidative stress. It was reported previously that FOXO3 is able to increase the expression of three FOXO transcription factors (FOXO 1, 3, 4) in a positive feedback loop influencing signaling pathways regulated by mentioned transcription factors (Roupe et al. 2014). In our experiments the up-regulation of *FOXO1* after P2 treatment was not observed.

### Cell Morphology

**Fig. 3:**
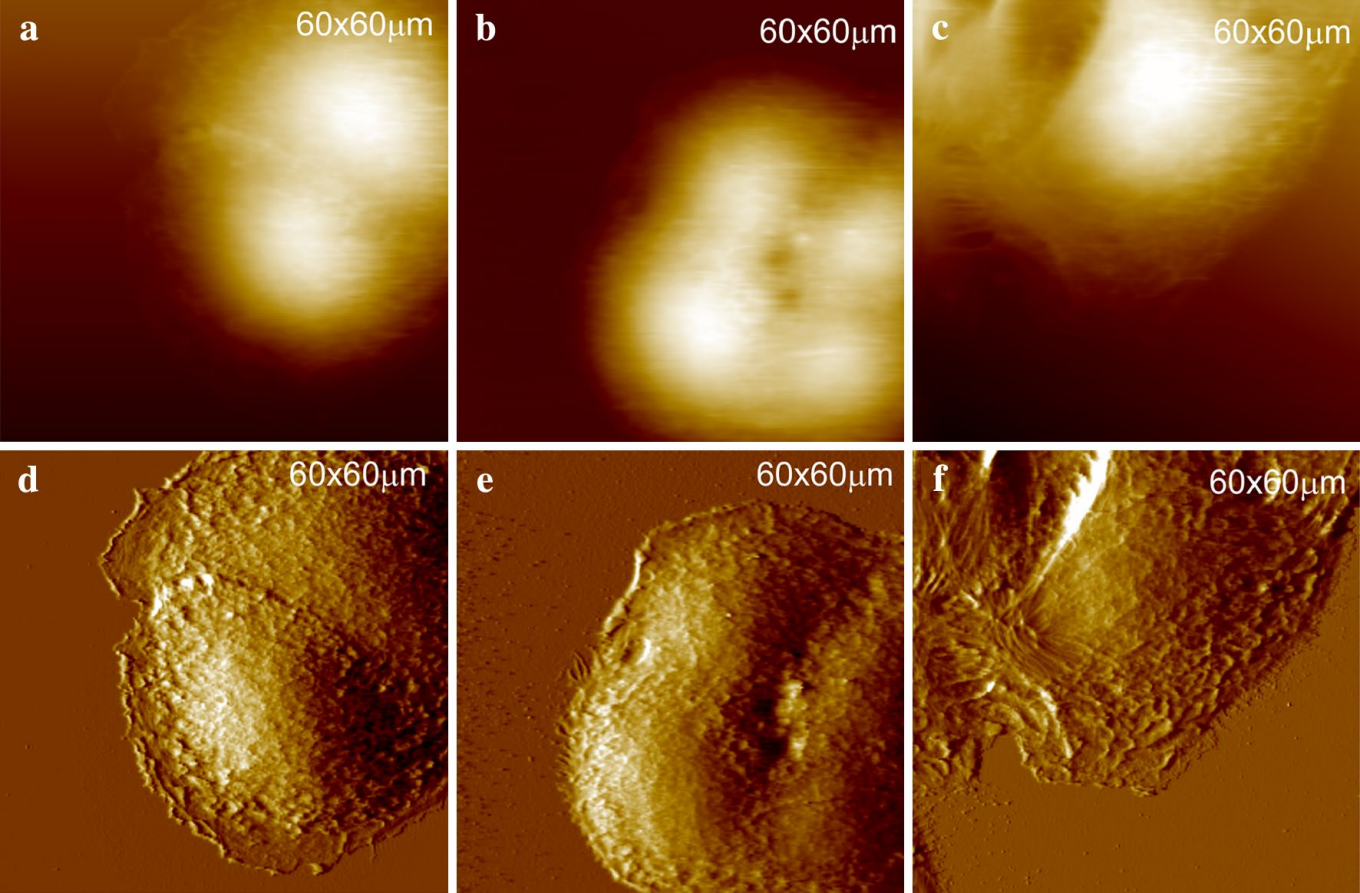
AFM images in contact mode topography of the studied HaCaT cells: height image of a typical control keratinocyte cell (**a**); height image of 0.5 μg/ml P1 treated cell (**b**); height image of 0.5 μg/ml P2 treated cell (**c**); deflection image of a typical control keratinocyte cell (**d**); deflection image of 0.5 μg/ml P1 treated cell (**e**); deflection image of 0.5 μg/ml P2 treated cell (**f**)

The AFM images of the HaCaT cell surface topography treated with P1 (Fig. 3b) and P2 (Fig. 3c) at 0.5 µg/ml concentration with untreated cells as the control (Fig. 3a) are shown.

Panels in the Fig. 3 demonstrate three sets of data consisting of two simultaneously captured frames of height images (a, b, c) showing overall topography and deflection images (d, e, f) showing details of this topography. It is clearly visible that cell treatment with 0.5 µg/ml of each peptide did not influence the cell topography. Key mechanical properties of cells are dependent on actin fibers organization (Ogneva 2013).The actin cytoskeleton has a fundamental role in various cellular processes such as migration, morphogenesis, cytokinesis, endocytosis, and phagocytosis. Therefore, we imaged the actin cytoskeleton distribution of untreated and treated HaCaT cells. Three non-toxic concentrations of each peptide were chosen based on viability assay. Fluorescence images showed different organizations of actin filaments dependent on a peptide type and concentration.

**Fig. 4:**
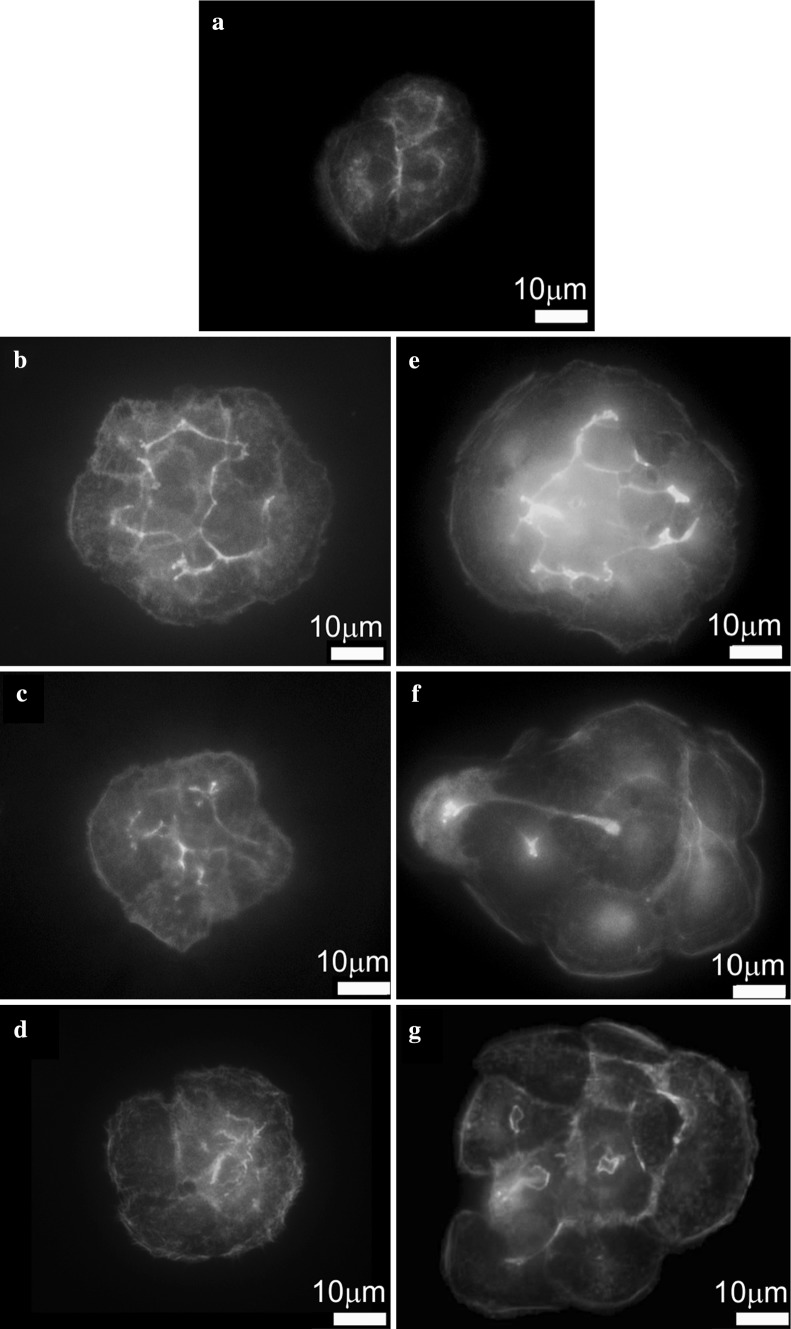
Fluorescence microscopy analysis of F-actin organization in HaCaT cells of: untreated cells (**a**); cells incubated with 0.5 μg/ml of P1 (**b**); cells incubated with 5 μg/ml of P1 (**c**); cells incubated with 50 μg/ml of P1 (**d**); cells incubated with 0.05 μg/ml of P2 (**e**); cells incubated with 0.5 μg/ml of P2 (**f**); cells incubated with 5 μg/ml of P2 (**g**)

Phalloidin staining of untreated cells shows actin localization predominantly at cell–cell junctions and dens actin mesh network mainly in the central cytoplasm region Fig. 4a. The exposure of the cells to investigated peptides after 48 h of treatment altered the actin filament distribution in a concentration depended-manner. Uniform distribution of the dense actin filament network in the cell after treatment with the P2 at lowest concentration (0.05 µg/ml) is noticeable (Fig. 4e). Nevertheless, the structure of individual filaments is not well distinguished. At higher P2 concentrations (0.5 and 5 µg/ml) rarefaction of actin filaments network occurred, especially in the peripheral parts of the cells, what can affect the cell stiffness (Fig. 4f, g). The actin in the cells treated with the highest concentration of P2 (5 µg/ml) was mainly in the form of tiny grains mainly concentrated in cell central part and in the cortex (Fig. 4g). These results are in agreement with *ACTN1* expression level that was slightly up-regulated in the cells threatened with the lowest (0.05 µg/ml) concentration of P2 (p < 0.05). In the cells treated with the highest concentration of P2 the actin expression level was significantly lower in relation to results obtained for 0.05 µg/ml of this peptide (p < 0.05). It was postulated by the manufacturer (Lipotec) that P2 is acting by imitating activity of FOXO3a (member of the forkhead box transcription factors). The FOXO3a acts as a trigger for apoptosis through up-regulation of genes necessary for cell death or down-regulation of anti-apoptotic proteins (Ekoff et al. 2007; Skurk et al. 2004). Moreover, down-regulation of FOXO3a is often seen in a various cancers. This protein is known as a tumor suppressor (Myatt and Lam 2007). It is known that FOXO3a is also involved in protection from oxidative stress by up-regulating antioxidant enzymes such as catalase and manganese superoxide dismutase. All these indications confirm maintaining the homeostasis of the cell and DNA protective role of FOXO3a. It was shown that P2 increased induction of DNA repair in primary human dermal keratinocytes transfected with a UVC-damage plasmid. Moreover, experiments presented by the producer revealed that this molecule decreased the number of senescent cells—primary human dermal fibroblasts from a 55-year old donor (Lipotec). These indications are also in agreement with investigations reporting that FOXO3 might be associated with the longevity of humans and animals (Willcox et al. 2008). It was also shown that FOXO3 knock out mice exhibit an age-dependent infertility, due to premature ovarian failure (Gallardo et al. 2007). In our experiments we demonstrated noticeable dose-dependent influence of P2 on actin organization in HaCaT keratinocyte cells that can induce reduction of cell rigidity confirming its potential application as anti-aging cosmetics ingredient. Moreover, we confirmed that P2 in the lowest tested concentration is able to up-regulate (p < 0.05) the expression of at least one of the mitochondrial detoxification enzymes (SOD2) (Fig. 2b).

The postulated role of P1 in the cell is different. P1 is responsible for enhancing production of key proteins Fibulin 5 (FBLN5) and Lysyl Oxydase-like 1 (LOXL1) required for the assembly of elastin. There are reports showing that cells treated with P1 produced more elastin, and collagen type I. Additionally, presented by the producer DNA microarray analysis revealed that this substance up-regulated collagen type I, XIV, IV and VI gene expression, as well as up-regulated also genes involved in cellular adhesion like: zyxin, integrin, talin and actin in human dermal fibroblasts (Lipotec). All these evidences indicate that P1 contributes to cellular cohesion due to Focal Adhesions (FAs), interface between the actin cytoskeleton and Extracellular Matrix (ECM) by overexpressing genes involved in these processes. Our experiments showed similar high density of actin filaments in the whole cytoplasm area, even in its peripheral parts, after treatment of the cells with the P1 at each tested concentration (Fig. 4b–d). Cells treated with P1 at the lowest concentration (0.5 µg/ml) revealed significant (p < 0.05) up-regulation of *COL17A1* gene.

### Elastic Modulus Determination

It has been demonstrated that modification of cytoskeleton organization leads to changes in mechanical properties of cells and these alterations can be investigated quantitatively (Safran et al. 2005). One option of determining the elastic modulus is to use the AFM, which measures the deformability of the cells in response to an applied force.

**Fig. 5:**
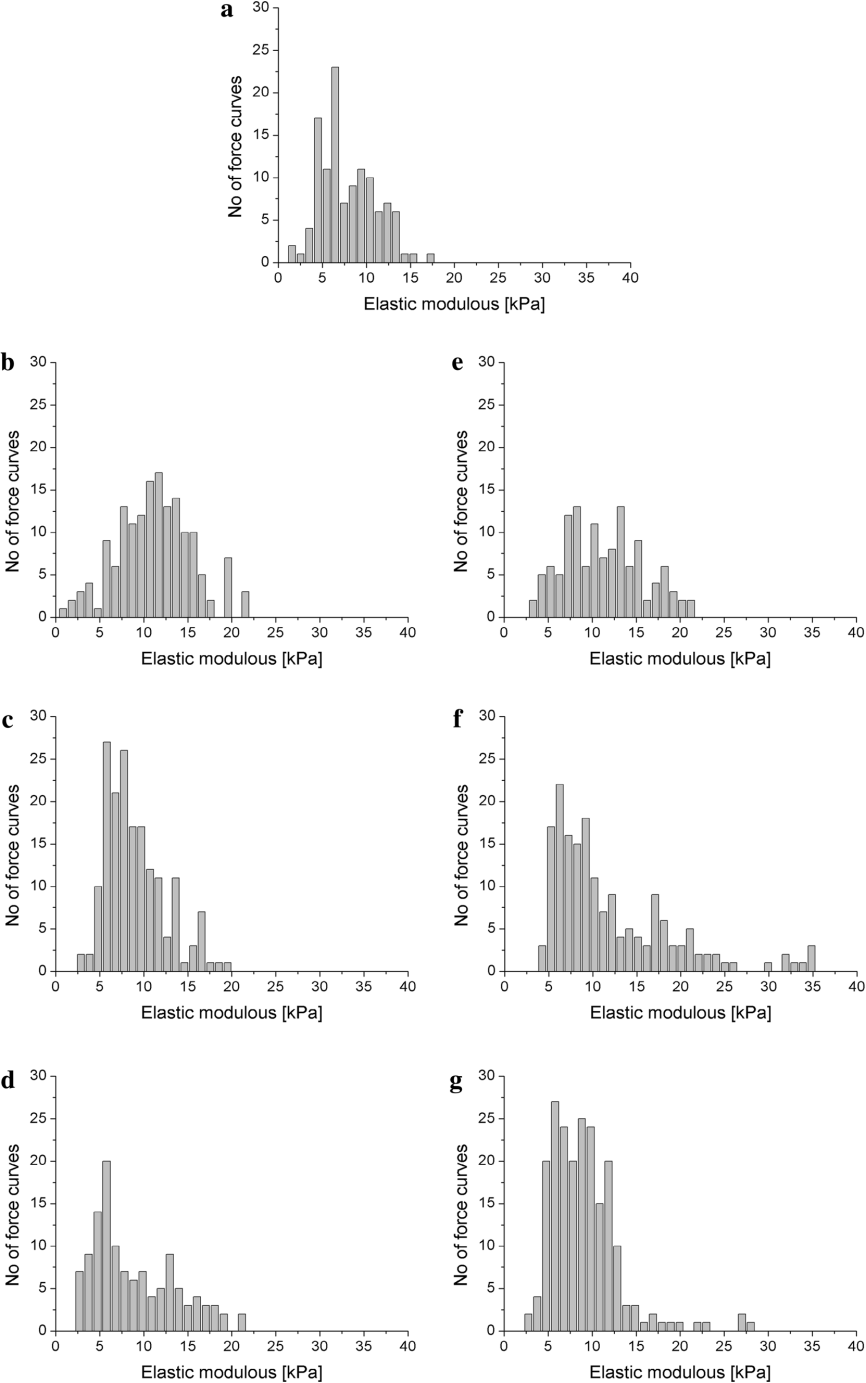
Histograms of the elastic modulus values of HaCaT cells measured for: **a** untreated cells as a control; **b** cells incubated with 0.5 μg/ml of P1; **c** cells incubated with 5 μg/ml of P1; **d** cells incubated with 50 μg/ml of P1; **e** cells incubated with 0.05 μg/ml of P2; **f** cells incubated with 0.5 μg/ml of P2; **g** cells incubated with 5 μg/ml of P2

The cells treated with the investigated peptides were the subject of our examination with reference to the untreated cells which served as the control. 200–300 individual measurements were carried out for each cell within the same region on its surface (around cell center) in order to obtain statistically significant results. The elasticity of the keratinocytes was calculated on the basis of force versus displacement measurements using an indentation depth of 200 nm, following a method described in the section Elastic Modulus of Keratinocytes. The distribution of determined elastic modulus values is presented in Fig. 5 in the form of histograms. Depending on the peptide and its concentration distinct characters of the elastic modulus distribution were observed. In case of control and the lowest concentration of peptides distribution of elastic modulus is symmetric and for higher concentrations becomes skewed to the right. The narrower distribution was observed for control and cells treated with peptide P2 at the highest (5 µg/ml) concentration.

**Fig. 6:**
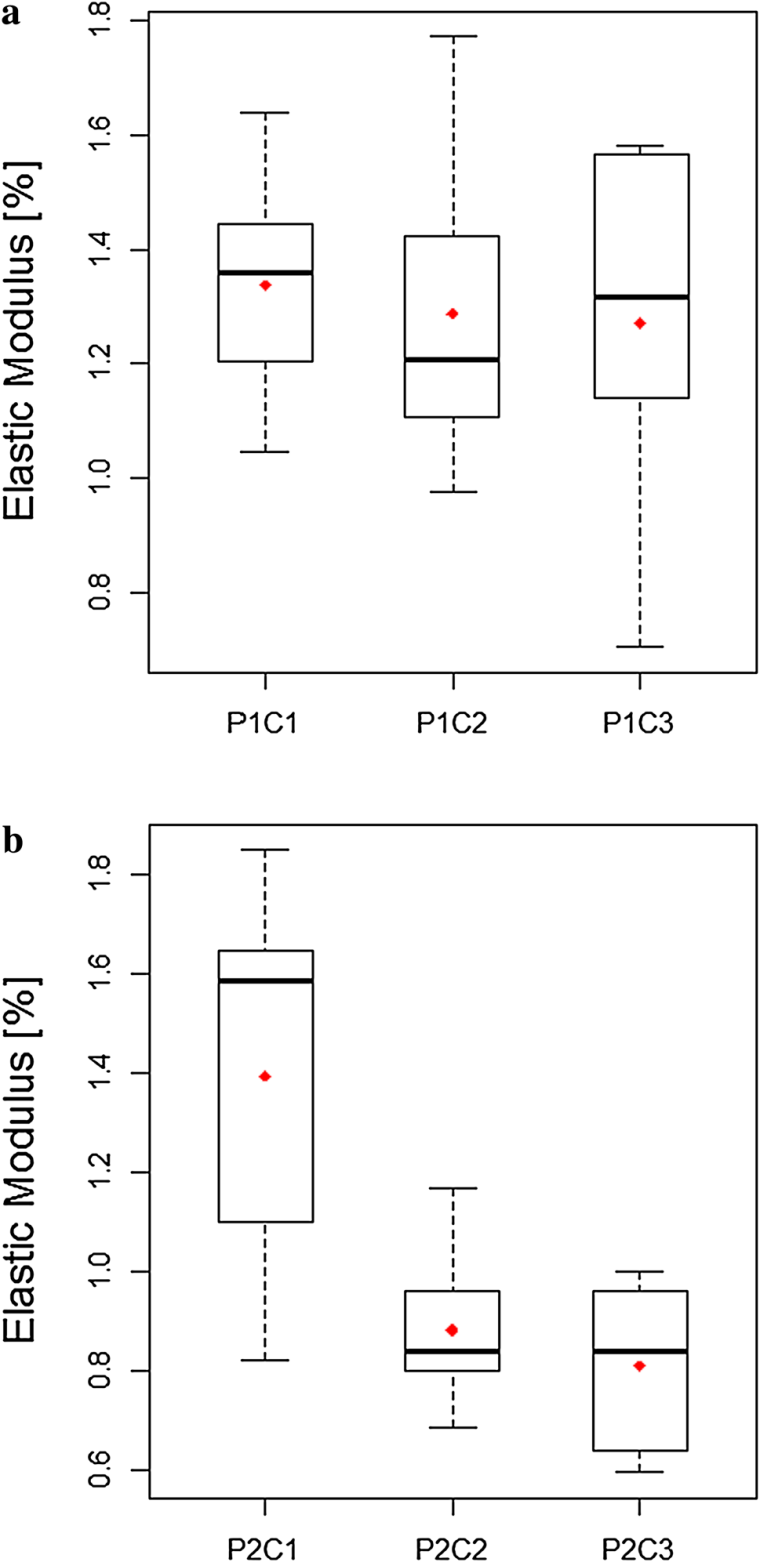
Relative changes in the elastic modulus values of HaCaT cells with respect to the control measured for cells treated with the peptide P1 **a** and peptide P2 **b**. The results are presented as boxplots: the top of each rectangle indicates the third quartile, a bold line in the middle of the rectangle indicates the median, the dot represents the mean value and the bottom of the rectangle indicates the first quartile. One vertical line (the top whisker) extends from the top of the rectangle to indicate the maximum value and the second one (the bottom whisker) extends from the bottom of the rectangle to indicate the minimum value

All calculated values of the elastic modulus were normalized to the value obtained for the control measurements and are presented in Fig. 6 as boxplots.

After the exposure to P2 at lowest concentration of 0.05 µg/ml (P2C1), an increase of the cell stiffness was noticed. On the other hand, the HaCaT cells treated with 0.5 µg/ml of P2 (P2C2) and with the highest (5 µg/ml) concentration of P2 (P2C3) showed the elastic modulus values lower than control, which corresponds to the alteration in the arrangement of actin filaments. Statistical analysis (Kruskal–Wallis test) revealed the significant differences (p < 0.05) between the P2C1 and higher concentrations of P2 (P2C2 and P2C3).

On the contrary, dosewise treatment with P1 caused increase of the HaCaT cells stiffness. Statistical analysis showed no significant differences between mean values of elastic modulus of HaCaT cells treated P1 in all considered concentrations (one way ANOVA, p > 0.05). It has to be noticed that different pathological processes, especially malignant transformation of cells, can cause changes in the mechanical properties resulting in the increase of the cell stiffness. This is connected with the impaired regulation of the actin cytoskeleton state (Ogneva 2013). In our experiments keratinocytes subjected to P1 treatment became more rigid than the control cells and ACTN1, COL17A1 and ITGB4 transcripts expression were down-regulated suggesting a disruption of cell mechanosensitivity homeostasis.

## Conclusions

In this work the studies of the influence of the two anti-aging peptides on the mechanical properties of keratinocytes cells were presented. It was pointed out that keratinocyte stiffness depends on the concentration of the investigated peptide. No significant differences in cells’ topography between untreated and treated cells were observed. On the other hand, exposure of HaCaT keratinocytes to P1 resulted in the increase of the cell rigidity. The most prominent effect was observed after incubation with 0.5 µg/ml of P1 and decreased gradually at higher concentrations. Simultaneously the expression level of three analyzed transcripts (ACTN1, COL17A1 and ITGB4) was appreciable down-regulated. P2 at lowest concentration of 0.05 µg/ml increased the density of actin filaments and stiffness of HaCaT cells what was correlated with higher ACTN1 transcript level. However, with raised concentration of P2 (0.5 and 5 µg/ml) rarefaction of actin filaments occurred simultaneously affecting the cell stiffness. The significantly lower modulus values obtained from the cells treated with P2 in above-mentioned concentration range indicate its potential application as anti-wrinkle cosmetics ingredient. In order to reveal the exact mechanism of action of both peptides, the whole genome expression studies by NGS technique would be required. However, at this stage, our results indicate that the quantification of mechanical properties of the skin cells seems to be a highly valuable tool in evaluation of topical peptides efficiency.
